# The need for fully-effective federated analytics of data sources for clinical trials

**DOI:** 10.23889/ijpds.v11i1.3201

**Published:** 2026-08-27

**Authors:** Stella Maris Fabiane, Sharon B. Love, David Fisher, Ian R. White, Jayne F. Tierney, Catherine Dampney, Folkert W. Asselbergs, Philip R. Quinlan, Macey L. Murray, Matthew R. Sydes

**Affiliations:** 1 UCL Innovative Clinical Trials Unit, Institute of Clinical Trials and Methodology, UCL, London, WC1V 6LJ, UK; ORCID: 0000-0001-5957-7275; 2 UCL Innovative Clinical Trials Unit, Institute of Clinical Trials and Methodology, UCL, London, WC1V 6LJ, UK; ORCID: 0000-0002-6695-5390; 3 UCL Innovative Clinical Trials Unit, Institute of Clinical Trials and Methodology, UCL, London, WC1V 6LJ, UK; ORCID: 0000-0002-2512-2296; 4 UCL Innovative Clinical Trials Unit, Institute of Clinical Trials and Methodology, UCL, London, WC1V 6LJ, UK; ORCID: 0000-0002-6718-7661; 5 Kent, Medway and Sussex Secure Data Environment, Kent, Medway and Sussex Secure Data Environment - NHS England Digital;ORCID: 0009-0004-1807-3758; 6 Institute of Health Informatics, University College London, London, NW1 2DA, UK; The National Institute for Health Research University College London Hospitals Biomedical Research Centre, University College London, London, NW1 2PG, UK; Department of Cardiology, Amsterdam Cardiovascular Sciences, Amsterdam University Medical Center, University of Amsterdam, 1105 AZ Amsterdam, The Netherlands; ORCID: 0000-0002-1692-8669; 7 School of Medicine, NIHR Nottingham Biomedical Research Centre, University of Nottingham, NG7 2UH, UK; ORCID: 0000-0002-3012-6646; 8 UCL Innovative Clinical Trials Unit, Institute of Clinical Trials and Methodology, UCL, London, WC1V 6LJ, UK; Transforming Data for Trials programme, Health Data Research UK (HDR UK), London, NW1 2BE, UK; ORCID: 0000-0001-6418-0854; 9 UCL Innovative Clinical Trials Unit, Institute of Clinical Trials and Methodology, UCL, London, WC1V 6LJ, UK; Data for R&D, “Data Access & Partnerships, Technology Data & Digital”, NHS England, London, SE1 8UG, UK; ORCID: 0000-0002-9323-1371

**Keywords:** Federated analytics, Health systems data, HSD, Clinical Trial, RCT

## Abstract

**Introduction:**

Clinical trials are usually analysed in a single environment allowing for flexible analysis including adjustment or stratification by subgroup: ‘one-stage’ analysis of individual-level data. Health systems datasets, often distributed across geography and providers, can streamline clinical trials. Data are increasingly accessible in secure data environments (SDEs). Future trial analyses may involve working across multiple SDEs. Row-level data and identifiable data often cannot leave, requiring a ‘two-stage approach’, where summary data from each SDE are meta-analysed.

**Objective:**

To quantify the potential loss of precision and concomitant increases in required sample sizes, and to make recommendations for trial design and conduct, if clinical trial data are split across silos (e.g. SDEs).

**Methods:**

Simulations used data from clinical trials in breast cancer, tuberculosis and prostate cancer with time-to-event, binary and continuous outcome measures. Silos were mimicked by 1000 random partitions into 2, 4, 10 and 25 equal silos and 4 unequal silos proportionate to the UK nations. Data were analysed as if the data could be pooled ignoring silo, pooled accounting for silo (one-stage) or not pooled (two-stage). Estimates and standard errors were presented graphically.

**Results:**

For all three outcome measure types, standard errors increased while point estimates spread out as more silos were introduced. Small biases occurred for binary and time-to-event outcomes. This did not always appreciably reduce efficiency. However, in one example with time-to-event data and the largest number of silos, a near-doubling of sample size would have been required to pre-emptively offset the loss of efficiency.

**Conclusion:**

Any need to use a two-stage analysis approach has a negative effect compared to doing a one-stage analysis. Technical and data governance solutions to support one-stage analyses are recommended.

## Introduction

Clinical trial datasets are usually collated and analysed in a single environment, allowing for flexible analysis including adjustment or stratification by subgroup. However, external factors are culminating in data being held in different environments, which could affect future design and analyses of trials.

Governance of sensitive data has evolved in response to digital and technological advances, with increasing emphasis on data security and protection, especially in health research. The Five Safes framework (safe data, projects, people, settings, and outputs) pioneered by the UK Office for National Statistics is widely adopted by data controllers in secure data environments (SDEs), also known as trusted research environments or data safe havens [1–4]. Following the Life Sciences Vision (2021) [5, 6] and Goldacre Review (2022) [7], there is now an accelerated move in the UK towards providing researchers access to health data safely via SDEs rather than distributing data to the researcher’s single location for a study. In SDEs, sets of defined data are securely provided by data controllers for appropriate analysis by validated ‘safe researchers’, which safeguard patient privacy and datasecurity.

Judicious use of high-quality data from health systems datasets (HSD) will streamline future clinical trials, facilitating planning, accrual and data collection [8–10]. Information about relevant individuals may be held across separate data locations, for example, multiple HSD of hospital admissions, primary care records and disease registries. Currently, clinical trial sponsors can collate the necessary HSD (‘bespoke extracts’) about their trial participants for detailed analysis of outcomes in their single environment [4, 11, 12]. In the near future in England, the default route to access these data is likely to be in SDEs, potentially based on geographical regions. SDEs operate with disclosure control processes in place, and individual level data (even if anonymised) often cannot easily egress an SDE. Therefore, a trial sponsor would likely be faced with the technical and governance challenges of performing HSD-based analyses in each environment separately and meta-analysing those results.

We sought to assess the impact on trial analyses if data are analysed in (or as if they are in) the same place. Where data can be pooled, the trial analysis becomes analogous to a ‘one-stage’ meta-analysis of individual participant data (IPD) [13], occasionally known as a ‘mega-analysis’ [14]. Any adjustment or stratification for clinical site or other grouping is straightforward and allows for a wide range of analysis possibilities. Where data cannot be pooled, we assumed isolated analyses would need to be done within each environment or silo, and the relevant summary data synthesised in a second analysis stage. This is analogous to a ‘two-stage’ meta-analysis approach, where the second stage is performed on aggregated data [15]. Hence, clinical trialists can learn from meta-analysts who are used to working across different clinical trials and, for various reasons, cannot always access IPD [16]. Meta-analysts usually prefer pooled IPD approaches over aggregated data for the depth and reliability of the analyses available, as well as simplicity [17].

We anticipated a two-stage meta-analytic approach may have an inflationary impact on the number of people required to participate in a study. Impact may be exacerbated by noncollapsibility, defined as “a failure of the measure when taken on a group to equal a simple average of the measure when taken on the group’s members or subgroups [18].” Theory suggests that siloing (splitting data) in noncollapsible settings would be more problematic (i.e. moving from a marginal estimand to one which conditions on silo) [19] and should lead to both larger magnitude of effect size *and* larger standard error, but similar significance levels[20].

The aims were to: explore any loss of precision if two-stage approaches were required; determine how to mitigate this upfront during sample size calculations; and elucidate longer term solutions.

## Methods

We needed several different siloed datasets with varying outcomes. As datasets that were already siloed across separate SDEs were not readily available, we conducted simulated analyses in large, existing datasets from three clinical data sources. Silos were mimicked with datasets partitioned as if they had been split across siloed environments.

### Data sources

We used relevant, limited, pseudonymised participant data from three clinical trials that took very different approaches. No HSD, participant identifiers or sensitive characteristics were requested. Where relevant, data access requests were approved following review from each trial’s data access committee. No further ethics committee approval was required. The German breast cancer study is a standard observational dataset for exploring time-to-event analyses provided by StataCorp [21]. The dataset includes recurrence-free survival time for 686 women with primary node-positive breast cancer, with 8 predictor variables. We used recurrence-free survival without the censoring variable as a continuous outcome, and with censoring as a time-to-event outcome. TB-IPD is a pooled, pre-harmonised dataset comprising IPD for randomised controlled trials of treatments for tuberculosis (TB) [22]. We used the 2021 dataset containing 17,507 participants from eight studies. The datasets included a binary outcome measure of cured or not, where ‘not’ includes all other disease stages. For the purposes of this analysis, the data are treated as if they come from one very large clinical trial. STAMPEDE is a multi-arm multi-stage platform trial for newly-diagnosed advanced prostate cancer; data were obtained from one comparison of 1176 participants who were allocated 2:1 to standard-of-care or standard-of-care plus docetaxel [23]. We used prostate-specific antigen (PSA) levels (ng/mL) collected 6 months after randomisation as a continuous outcome, and overall survival as a time-to-event outcome. The primary outcome measures in the original analysis were overall survival in STAMPEDE and recurrence-free survival time in the German breast cancer dataset. The results of our illustrative analyses should not be used to inform clinical practice.

### Mimicking silos

Each dataset effectively comprised IPD in a single analysis environment. To mimic siloing, we partitioned each dataset following three approaches. First, the participants were partitioned randomly across 2, 4, 10, or 25 equally sized silos. Second, the participants were partitioned randomly across unequal silos of size approximately 84%, 8%, 5% and 3%, proportional to the populations of England, Scotland, Wales and Northern Ireland, the four nations of the UK. This scenario was to explore the implications on UK-wide trials if approaches to data access do not permit interacting with the data in one place. Finally, we randomly partitioned the participants into nine silos of unequal sizes of 16%, 16%, 13%, 11%, 11%, 10%, 10%, 9% and 5%, approximately proportional to the populations of nine English regions defined by Office for National Statistics (ONS). [24] In each scenario, the random partitioning was repeated 1,000 times.

### Statistical analysis

Our ‘reference’ analysis was performed by fitting the analysis model to the full data set, without adjustment for silo. This represents the ideal situation of a single analysis environment where the source of siloing could be ignored, and is typical of how clinical trial sponsors have historically interacted with data. A variation on this analysis additionally adjusted for silo as an unordered categorical variable in the model: we refer to this as ‘one-stage’, where silo is equivalent to study in a one-stage IPD meta-analysis. To mimic instances where data cannot be pooled across silos, we performed a ‘two-stage’ analysis by separately fitting the analysis model to the data available in each silo, extracting the estimated treatment effect and standard error. We combined these estimates using a common-effect meta-analysis [25] to provide the overall estimate. Where the treatment effect represented an odds ratio or hazard ratio, the regression coefficients (i.e. the log odds ratio or log hazard ratio) were used for combining across trials. We did not allow for heterogeneity between silos since primary clinical trial analyses do not usually allow for treatment effect heterogeneity across geographical settings, and since silos will typically be subject to the same protocol.

For the German breast cancer study, we illustrated a collapsible analysis by fitting a linear regression of recurrence-free survival time on hormonal therapy, adjusted for the other baseline covariates, ignoring censoring. We illustrated a noncollapsible (time-to-event) analysis with a Cox regression on hormonal therapy, unadjusted and adjusted for age and menopausal status. In each case, the estimand of interest was the coefficient of hormonal therapy, representing the mean difference in survival times assuming no censoring and the log hazard ratio for recurrence or death. For TB-IPD, the analysis of interest was a logistic regression of presence or absence of cure on culture status, age, sex, body mass index, HIV status and smear status at baseline, plus time to culture conversion. The estimand of interest was the coefficient of culture status. We refer to culture status as ‘treatment’ to be consistent with the other data sets. Our covariate of interest was non-randomised in both these datasets, so the coefficients only have causal interpretation under a no unmeasured confounders assumption: however, our comparisons are between analyses adjusting for the *same* confounders, so any residual confounding does not affect the comparisons.

For STAMPEDE a linear regression analysis was done with the outcome being log transformed prostate-specific antigen (PSA) at 6 months post-randomisation. The covariates were treatment, log PSA at randomisation, age dichotomised at 70 years, and presence of metastasis, with 714 observations available. The estimand of interest was the coefficient of docetaxel treatment. We also analysed a second outcome measure, time from randomisation to death or last follow-up. Covariate adjustment is more unstable in smaller datasets, so we explored this analysis both with and without covariate adjustment. Hence, two Cox regressions on treatment arm were done: one adjusting for nodal stage, planned radiotherapy, age dichotomised at 70 years, WHO performance status at randomisation, metastatic status, regular NSAID use at baseline, and trial-specific time period; the other without adjustments.

We examined whether there were systematic differences in standard errors between analysis approaches, broken down by outcome measure type, and whether there was evidence of ‘bias’ (i.e. systematic difference) in effect size between analysis approaches, also broken down by outcome measure.

We show the results graphically in Figures 1–7. For the reference analysis there are no silos. For ease of visualisation, results of 100 random partitions (10% randomly selected from 1000 simulations) are displayed. The axes do not include zero. Monochrome versions of these graphs are given in the supplementary file.

**Figure 1:**
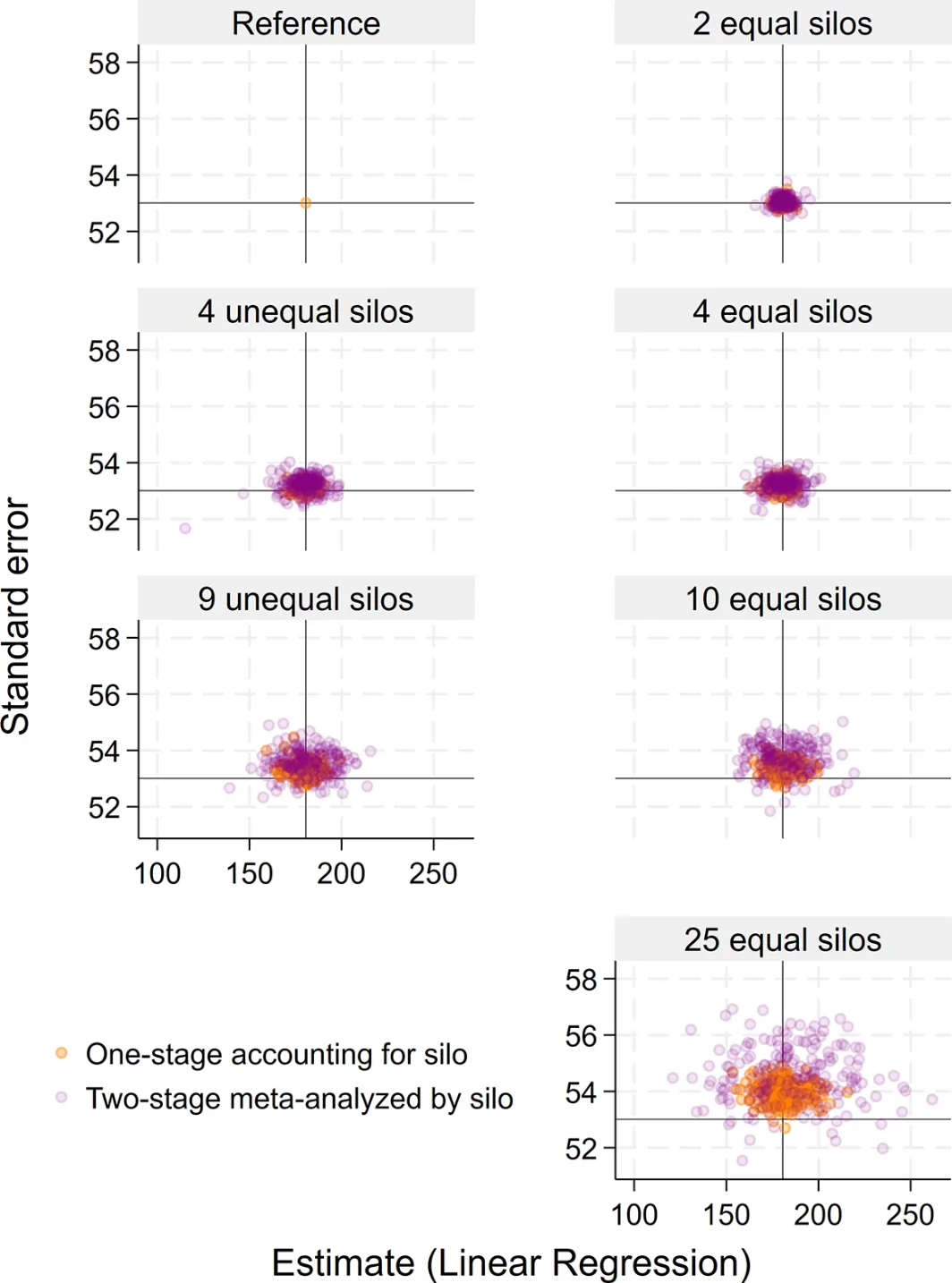
Individual participant data meta-analysis (one-stage) and aggregated data meta-analysis (two-stage) results for the continuous outcome, recurrence-free survival time ignoring censoring on hormonal therapy adjusted for age and menopausal status, from the German breast cancer study, using linear regression

**Figure 2:**
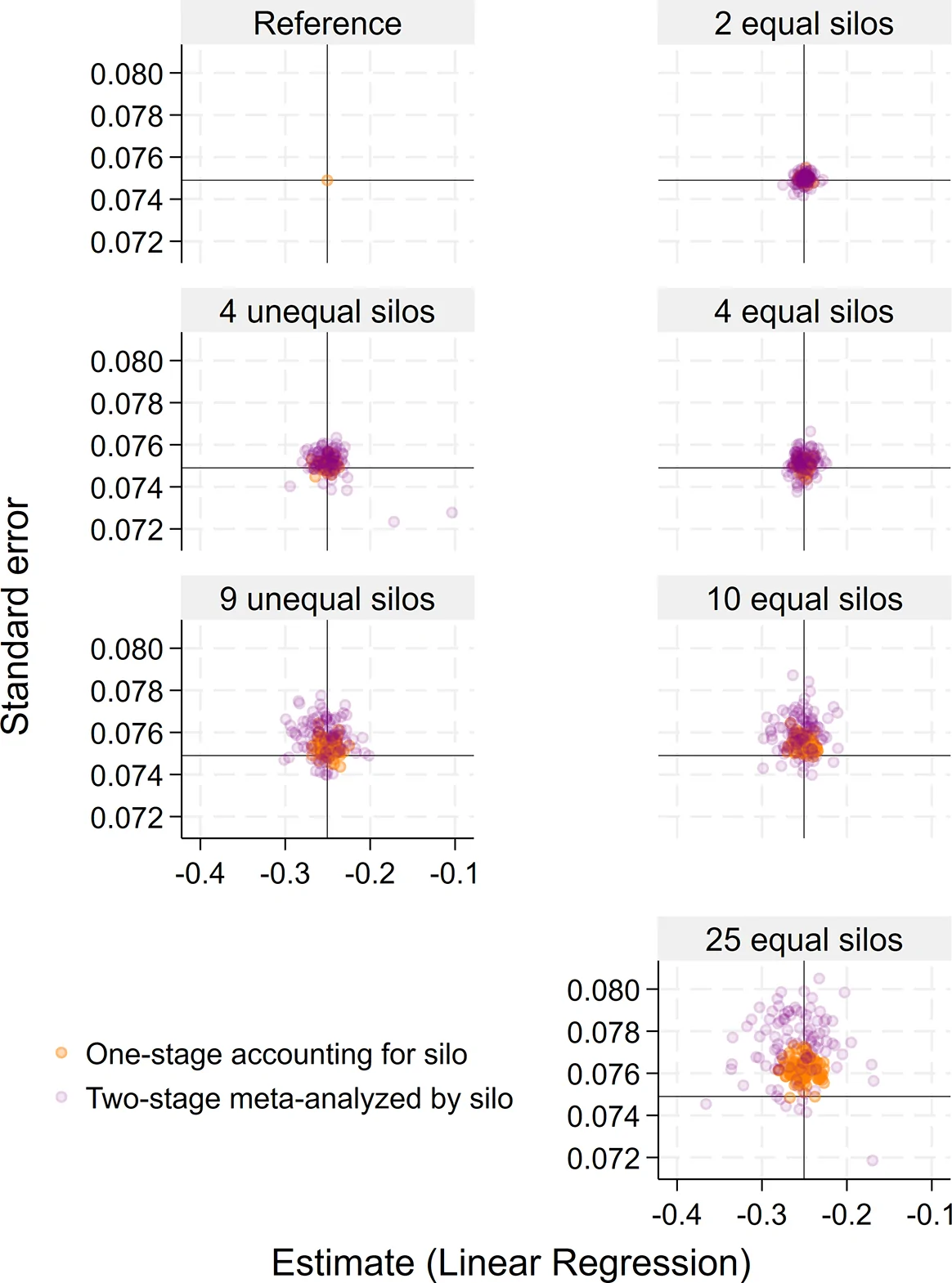
Individual participant data meta-analysis (one-stage) and aggregated data meta-analysis (two-stage) results for the continuous outcome, prostate-specific antigen level at 6 months post-randomisation, adjusted for log(PSA) at randomisation and age (dichotomised at 70 years) from STAMPEDE, using linear regression

**Figure 3:**
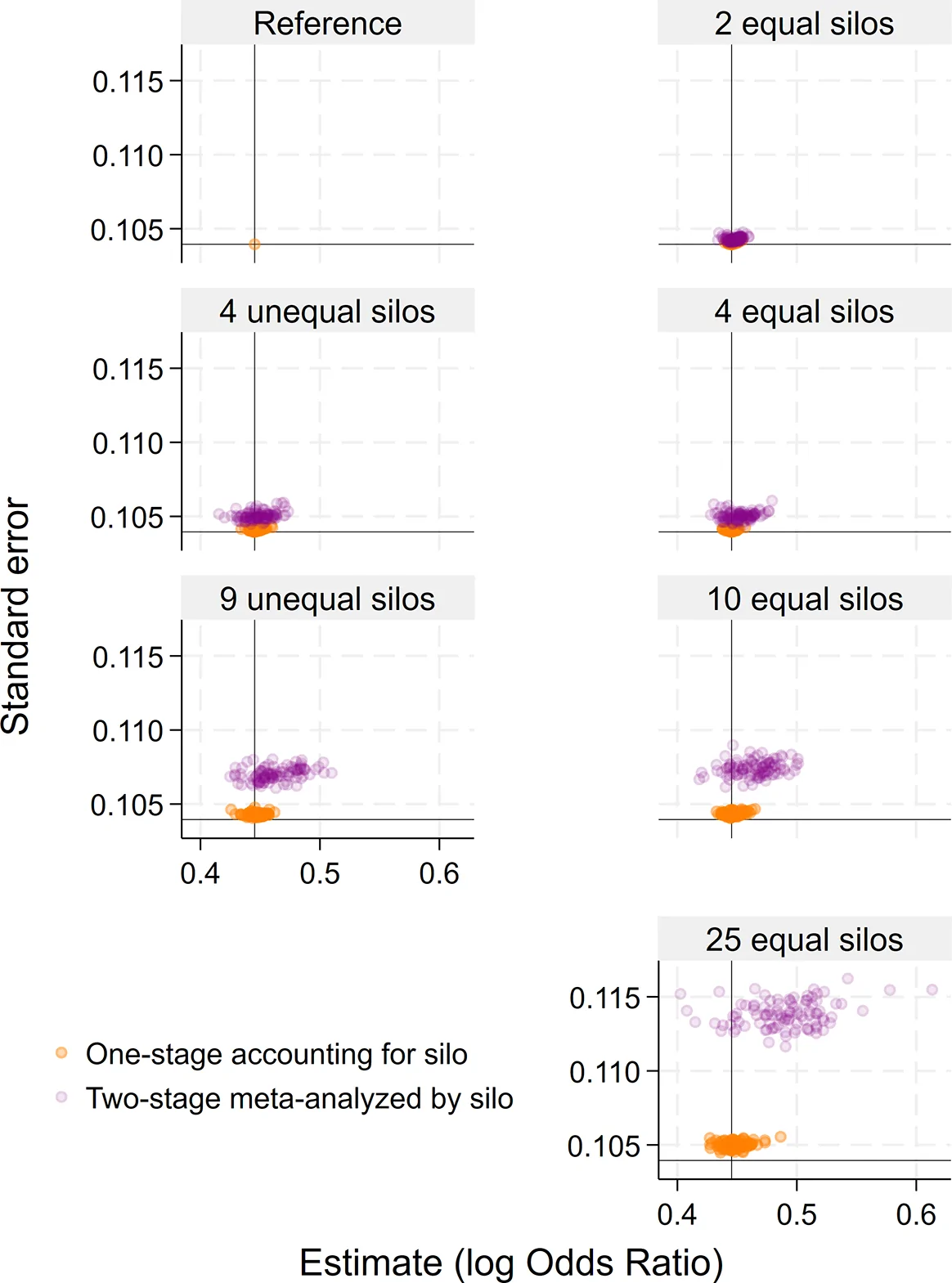
Individual participant data meta-analysis (one-stage) and aggregated data meta-analysis (two-stage) results for the binary outcome, presence or absence of cure from TB-IPD adjusted for culture status, age, sex, body mass index, HIV status and smear status at baseline, plus time to culture conversion, using logistic regression

**Figure 4:**
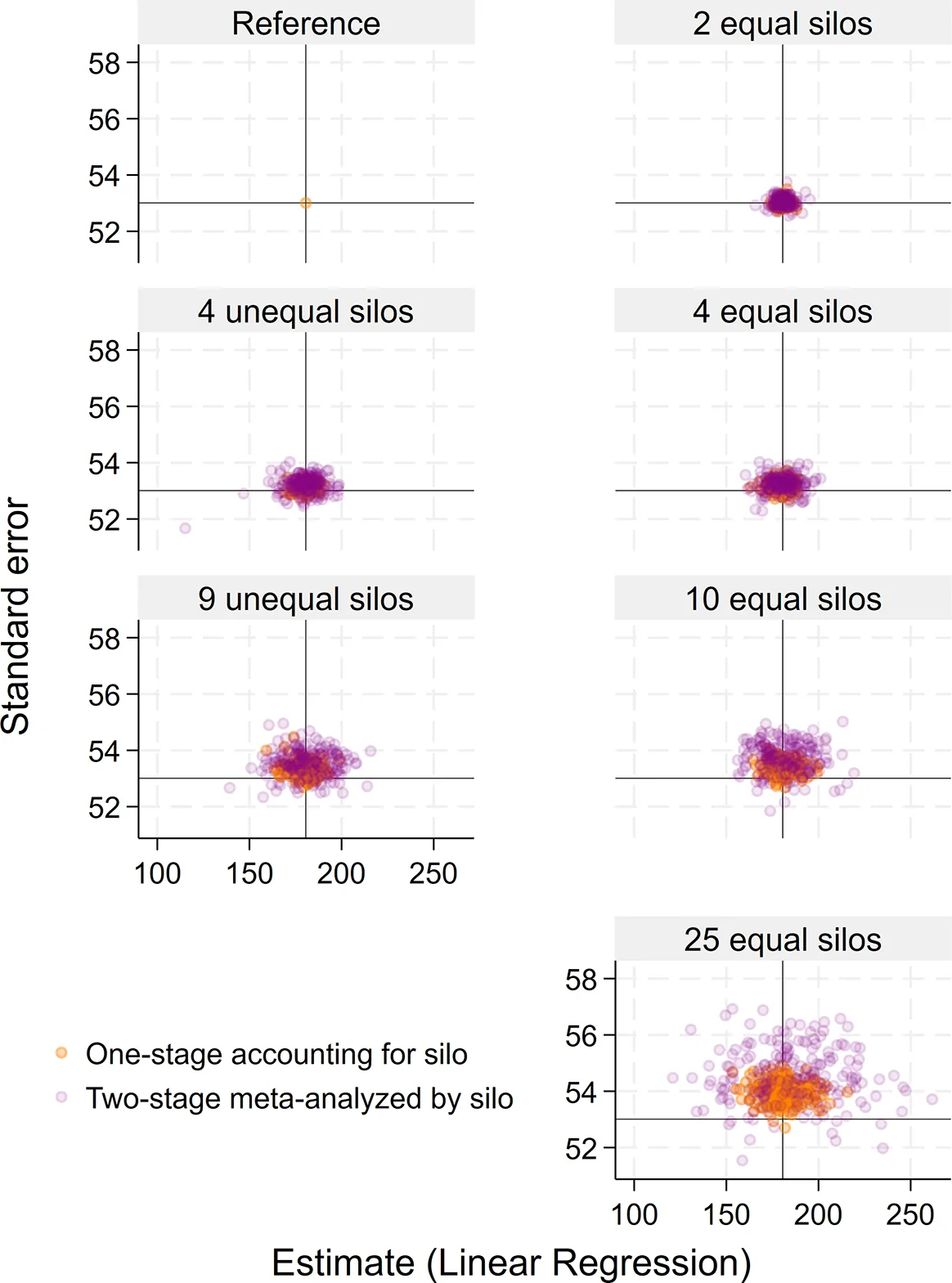
Individual participant data meta-analysis (one-stage) and aggregated data meta-analysis (two-stage) results for the time-to-event outcome of recurrence-free survival time on hormonal therapy from the German breast cancer study, using Cox regression adjusted for age and menopausal status

**Figure 5:**
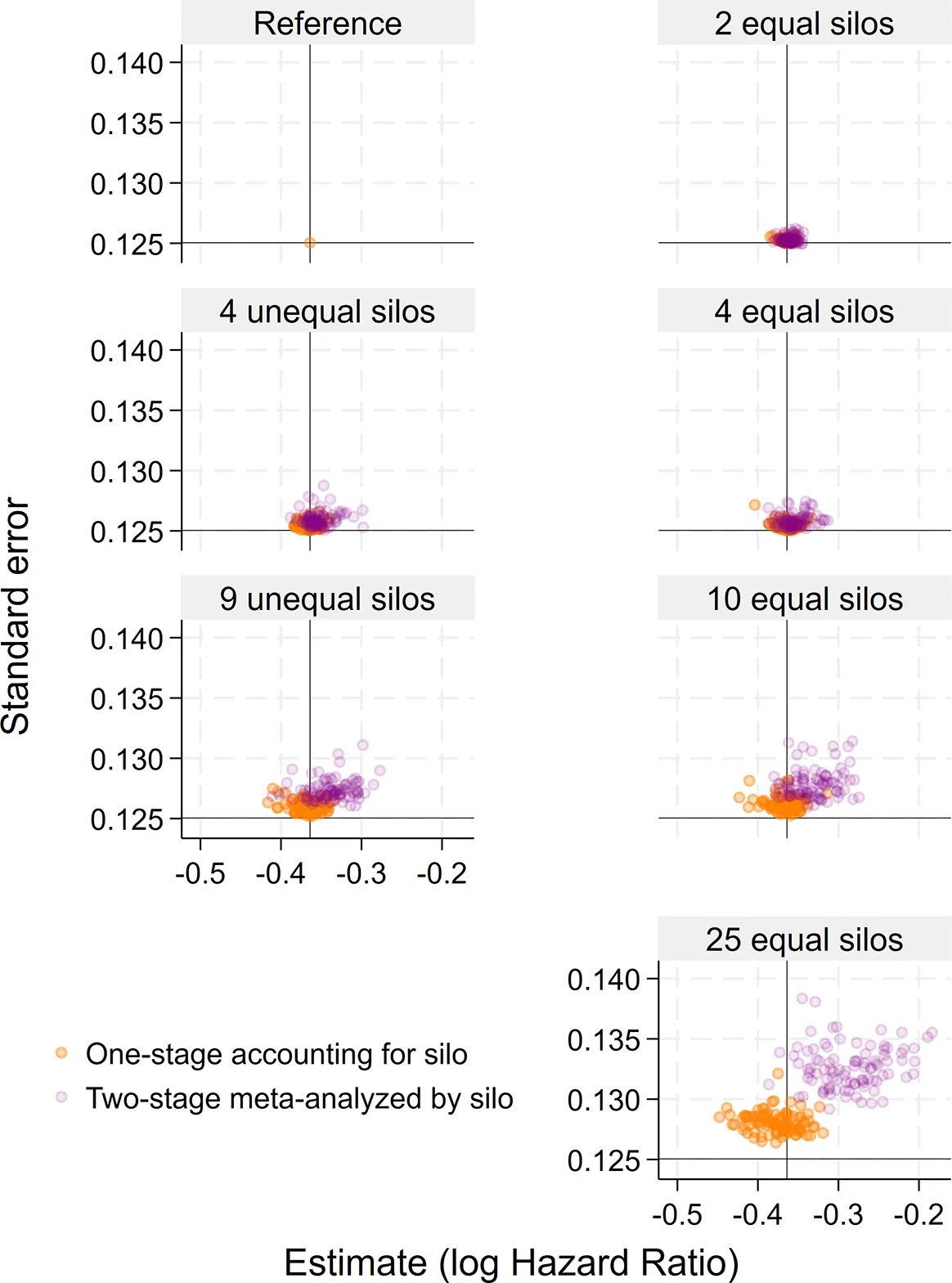
Individual participant data meta-analysis (one-stage) and aggregated data meta-analysis (two-stage) results for the time-to-event outcome of recurrence-free survival time on hormonal therapy from the German breast cancer study, using unadjusted Cox regression

**Figure 6:**
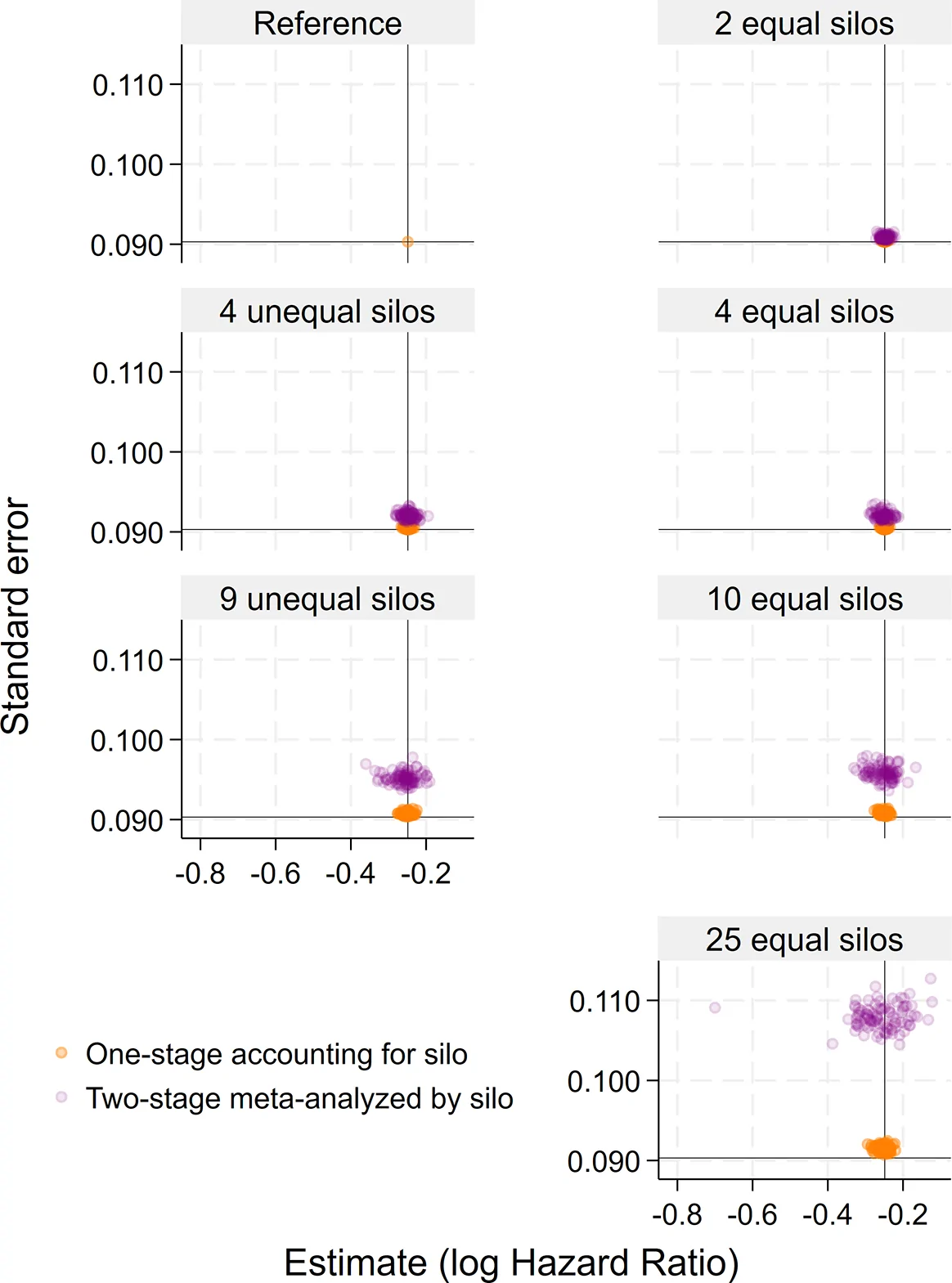
Individual participant data meta-analysis (one-stage) and aggregated data meta-analysis (two-stage) results for the time-to-event outcome of overall survival from STAMPEDE, using Cox regression adjusted for nodal stage, planned radiotherapy, age dichotomised at 70 years, WHO performance status at randomisation, metastatic status, regular NSAID use at baseline, and trial-specific time period

**Figure 7:**
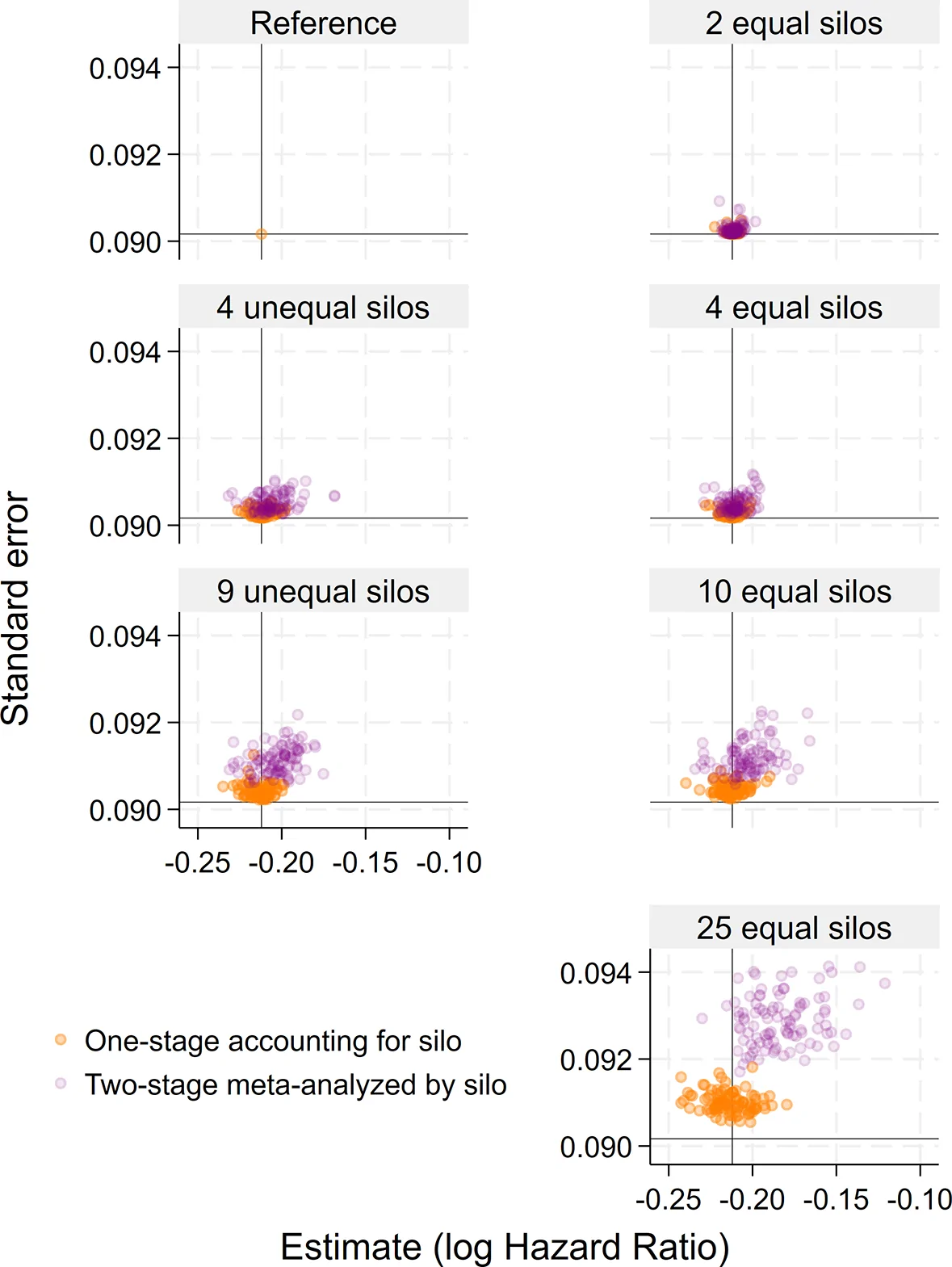
Individual participant data meta-analysis (one-stage) and aggregated data meta-analysis (two-stage) results for the time-to-event outcome of overall survival from STAMPEDE, using unadjusted Cox regression

### Sample size requirements

We used the effect sizes and their standard errors to estimate the increase in sample size that would be required for any clinical trial that used a one-stage or two-stage analysis (using silos) compared to a reference analysis (without silos). Following Julious (2004), [26] we tested the general null hypothesis f(*μ*) = 0 with two-sided Type-I error *α* using a test statistic S∼N(f(μ),Var(S)). The sample size requirement *N* to achieve Type-II error *β* under the alternative f(*μ*) = d is given by solving $Var(S)=\frac{d^{2}}{{\left(Z_{1-\beta }+Z_{1-\alpha /2}\right)}^{2}}$, where *N* is implicit in *Var*(*S*).

We have two tests *S*_1_ and *S*_2_ conducted on the same data set, where *S*_1_ is the reference analysis and *S*_2_ is either the one-stage or the two-stage analysis. We assume *Var*(*S*_1_) and *Var*(*S*_2_) are both inversely proportional to sample size *N*. The target effects for the two tests are *d*_1_ and *d*_2_, which differ for noncollapsible effect measures. Then the ratio of required sample sizes for test 2 compared with test 1 is given by


\frac{{Var(S_2)}/{d^2_2}}{{Var(S_1)}/{d^2_1}}.

Intuitively, this is the squared ratio of expected Z-statistics. For the reference analysis, we take *Var*(*S*_1_) as the squared standard error of *S*_1_ and *d*_1_ as the value of *S*_1_. For the one-stage and two-stage analyses, which vary across repetitions, we take *Var*(*S*_2_) as the average squared standard error of *Var*(*S*_2_) and *d*_2_ as the unweighted average value of *Var*(*S*_2_), taken across the 1,000 random partitions. Monte Carlo errors were computed using an extension of the methods used by the package simsum [27].

The analysis was carried out using Stata version 19.5 [28]. The code is given in the Supplementary Material.

## Results

Each analysis of the three trials is shown below as a figure with tabulated summary information in the supplement, presented in outcome measure order. In each instance, the point estimate and standard error (SE) is shown for the reference analysis (intersection of the reference lines) and then overlaid for the one-stage (orange circle) and two-stage (purple circle) meta-analysis approaches. To simplify visualisation, results of 100 partitions (10% randomly selected from the 1,000 simulations) are displayed.

For the continuous outcome measure using linear regression in the breast cancer data, the one-stage analysis found the coefficient of hormonal therapy to be 180.6 days, with a standard error (SE) of 53.0 days. Figure 1 and Supplementary Table 1 show results of the one-stage analyses adjusted for silo and the two-stage analyses (purple circles), across different patterns of silos. For the two-stage analyses, the SEs generally increased while the estimates spread out as the number of silos increased.

Using the STAMPEDE prostate cancer dataset for the continuous outcome measure of log PSA value at 6 months, the estimate under the reference analysis was -0.25 and SE 0.07. The pattern of distribution of the estimates and SEs was similar to Figure 1 (Figure 2 and Supplementary Table 2).

For the binary outcome measure in the TB-IPD dataset, analysed with logistic regression, the reference analysis gave an estimated log OR of 0.45 with a SE of 0.10. As the number of silos increased for two-stage analysis, as well as the spread of the estimates and the level of the SEs increasing, the mean of the estimates also increased away from the null (see Figure 3 and Supplementary Table 3).

For the time-to-event analysis using the breast cancer data, the reference analysis gave a log hazard ratio of -0.38 with SE 0.13 (-0.36 and 0.13 respectively for the unadjusted analysis). As the silos were introduced and increased in number, the SEs increased (Figures 4 and 5 and Supplementary Tables 4 and 5); the same effect was observed as for the logistic regression. Changes in point estimates and standard errors were similar to those seen in Figure 3. However, in Figures 4 and 5 the positive shifts in point estimates represents a shift towards the null, whereas in Figure 3 the shift was away from the null. The spread of values is larger for the two-stage analyses compared to the corresponding one-stage ones with the impact most clearly observed with 25 silos.

For the STAMPEDE time-to-event analyses, the same pattern of analyses was followed for the time-to-event Cox regressions using breast cancer data. The reference adjusted analysis had its estimate as HR=0.78, with a standard error of 0.07. The mean point estimate does not change in Figure 6 and Supplementary Table 6, unlike Figure 3 where it goes away from the null and Figures 4, 5 and 7 (and Supplementary Table 7) where it goes towards the null.

Table 1 and Figure 8 present the indicative modifications to sample size that would have been required to address the observations in the previous examples. Accounting for silo in a one-stage approach, or in a two-stage approach with continuous or binary outcome, would generally require minimal change in sample size. However, a two-stage approach may require substantial inflation under Cox PH regression especially if, as is standard, adjusting for baseline characteristics. With ten silos, increases of 24% and 9% would have been required in the breast cancer and prostate cancer examples, and this was much higher with more silos. Each would require a longer trial period and/or faster accrual rates.

## Discussion

For all three outcome measure types, when more silos were used, standard errors increased while point estimates spread out, indicating loss of efficiency. The loss was greater if the participants were distributed across more silos and less evenly. This reduction of efficiency could be offset by inflating the target sample size.

We have shown that any analysis using a two-stage meta-analysis approach due to partitioning of data can be much less efficient compared to being able to do a one-stage approach where all the data contribute simultaneously to analytic models. The spread of estimated treatment effects is larger for the two-stage analyses compared to the corresponding one-stage analysis, with the impact most clearly observed with 25 silos. This problem is more pronounced for more complex outcome measures such as time-to-event(survival).

This loss of efficiency induced by a two-stage approach could be nominally offset by inflating the target sample size of the trial. The inflation factor was <10% for continuous or binary outcomes and for time-to-event outcomes with fewer than 9 silos, but reached 7-24% for time-to-event outcomes with 9 or 10 silos and 35-91% for time-to-event outcomes with 25 silos. We explored a variety of silo sizes, and we anticipated that small silos may, by chance, lead to more extreme estimates of the treatment effect. The nature of the problem was greater if the participants were split more widely and less evenly. This would have substantial implications for many clinical trials. Many trials pre-emptively inflate their sample size in anticipation of issues with follow-up [29]. The inflationary factor required because of a two-stage analytic approach would be separate to this and would be multiplicative rather than additive. For example, a phase III trial requiring 500 people (to observe 200 events) might be inflated by 10% to 550 to account for anticipated loss to follow-up and a further 20% to 660 to account for a two-stage analysis. Therefore, such required inflation in sample size may offset the streamlining gained by using HSD.

In binary outcome measures, two-stage analysis biased the estimated treatment effect away from the null (Figure 3), probably due to noncollapsibility of the odds ratio. In time-to-event data, two-stage analysis could instead bias the estimated treatment effect towards the null (Figures 4, 5 and 7), even though noncollapsibility of the hazard ratio would suggest a bias away from the null. We believe this bias arises from failure of the Normal approximation when the silo-level data become sparse (few events). [30] These biases need to be taken into account when comparing methods, but the relative biases seen in Figures 3–7 were of limited clinical importance, being between 10% away from the null and 8% towards the null, except with 25 silos in Figures 4, 5 and 7 where they were 13-23% towards the null (Supplementary figure 1).

Use of a two-stage meta-analysis approach also complicates the statistical analysis. Subgroup analyses, whether pre-specified or exploratory, may be impacted because data within a subgroup within a silo will be particularly sparse. Data separated by silos also raises challenges in forming appropriate imputation models to handle missing data [31].

If the data for participants are held in separate locations, some duplication of effort in information governance is likely, regardless of whether the data can be analysed together or only separately. For data held in multiple environments, resolution will be needed for archiving according to the relevant legislation and reconstructing datasets for secondary or tertiary data reuse projects. Better data federation is associated with closer alignment, harmonised governance and improved trust [32].

**Table 1:** Sample size inflation required to offset loss of efficiency for one-stage and two-stage individual participant data analyses across silos, compared to analysing all data together without siloing

**Data**	**Outcome model**	**Approach**	**Equal silos**					**Unequal silos**	
			**2**	**4**	**10**	**25**		**4**	**9**
**Continuous**									
Breast Cancer	Linear regression	1-stage	0%	0%	1%	3%		0%	2%
		2-stage	0%	0%	1%	-1%		0%	0%
STAMPEDE	Linear regression	1-stage	0%	0%	1%	4%		0%	1%
		2-stage	0%	-1%	0%	0%		1%	0%
**Binary**									
TB-IPD	Logistic regression	1-stage	0%	0%	0%	0%		0%	0%
		2-stage	0%	0%	-1%	0%		0%	-1%
**Time-to-event**									
Breast Cancer	Cox PH adjusted	1-stage	0%	0%	0%	-1%		0%	0%
		2-stage	3%	8%	24%	91%*		8%	21%
	Cox PH unadjusted	1-stage	0%	0%	0%	-1%		0%	0%
		2-stage	3%	8%	24%	91%*		8%	22%
STAMPEDE	Cox PH adjusted	1-stage	0%	0%	0%	0%		0%	0%
		2-stage	1%	1%	9%	35%*		3%	7%
	Cox PH unadjusted	1-stage	0%	0%	0%	-1%		0%	0%
		2-stage	1%	2%	11%	42%*		4%	10%

Monte Carlo standard errors are 6-11% for results marked * and at most 4% for all other results. Cox PH -- Cox proportional hazards regression model.

**Figure 8:**
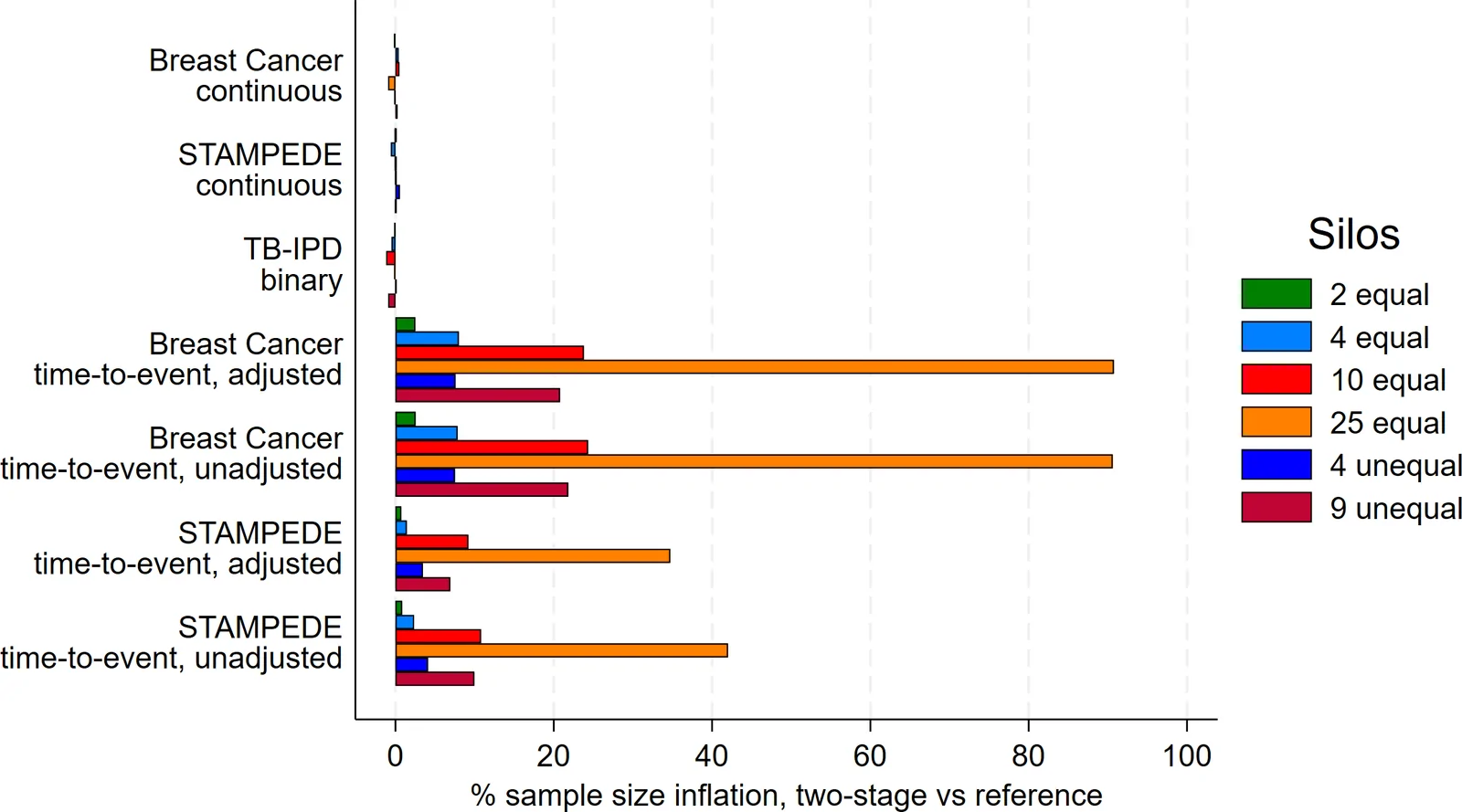
Sample size inflation for two-stage individual participant data analyses across silos, compared to analysing all data together without siloing

For all of these reasons, the conclusion reached for clinical trials is the same as that for meta-analysis: it is strongly preferable to interact with the data in a one-stage trial approach, where all the data are either actually in, or can be treated as if they are in, one place and included in the analysis model: preventing the problem is better than curingit [33].

The move towards ‘data access’ rather than ‘data sharing’ aims to restrict access to sensitive HSD [34]. SDEs are a safe and appropriate way to access information, particularly for projects where the participants have not given explicit consent, as in many retrospective real-world data projects [35]. For most clinical trials, there is an opportunity to obtain explicit consent which can include consent for the relevant, minimised data to be made safely available for analysis in the sponsor’s secure environment. Consistent forms of wording are required so data providers can egress datasets directly to trial sponsors for analysis [36].

One limitation of our work is that we did not access real HSD located in SDEs. Instead, entire clinical trial datasets were accessed and partitioned randomly to mimic having come from separate SDEs. However, this is also a strength: by creating many partitions of each dataset we were able to learn more about the broad pattern of what might happen, and to estimate a distribution of power loss. Previous authors have compared one-stage and two-stage approaches by simulating multiple trials [37], and by examining multiple different estimated quantities across the same four large trials [38]. However, their main objectives were to compare analysis approaches rather than to investigate the issue of siloing itself, and they did not examine time-to-event outcomes in which we have identified additional issues. Future research could explore a larger, more varied selection of real-world datasets, or a non-random partitioning mechanism.

The exact balance of participants across locations and SDEs for any particular trial may not be easily estimated up front. However, use of data in SDEs for trial planning, as well as delivery-through-data, should give a much better estimate of the actual numbers of participants that might be recruited and feed into sample size adjustment calculators.

The loss of efficiency due to siloing is not unique to clinical trials, but it is a particular issue for trials and other forms of prospective research predicated on *a priori* planning for a fixed number of participants to be recruited. For clinical trials with suitable participant consent, datasets could still be egressed from the relevant data providers, which may be multiple and shared in a safe and secure way to a safe and secure location from the trial sponsor, where they can interact with the data in one model. Alternatively, a full federated approach should be sought whereby the relevant, minimised datasets are pooled in one SDE where the trial sponsor’s representatives can interact with them in analysis. If pooling copies of datasets in one location is not possible, perhaps for reasons of information governance or file size, it will be preferable if technology were widely available to allow analysts to interact with the data as if they were in one place for one model, even though the datasets are actually in separate places. Some work is being started along these lines. Pilot studies run by TRE-FX and TELEPORT, supported by DARE UK, have developed approaches and technical solutions to federated analytics of genomic and health data [39, 40, 41]. Appropriate transformation of data assets into common data models may facilitate federated analytics; limitations in the mapping processes leading to data exclusion and loss of information have been reported [42, 43]. There are emerging technical approaches available for federated analytics although these have potential for statistical inefficiency. This work helps to demonstrate some benefits and risks of this mechanism and can help frame the utility of the technical innovation to support data that is held across multiple SDEs. There is still more to do to ensure full interaction with data, particularly with respect to clinical trials.

One response is for sponsors to continue with trial-specific data collection, an approach which is well understood, traditional, but duplicative of effort. Another is for development of new approaches to analysis that might mitigate the challenge of two-stage analysis across silos. A final response is for data providers to understand the implications of this work in order that they may sooner provide resolution towards one-stage analysis equivalent to pooling data in one place. In the NHS Research SDE Network, a network of geographically-defined SDEs with complete coverage in England and one national secure data environment, work towards full federation is ongoing. Multinational solutions are required. Many UK-wide trials will recruit from across regions covered by different SDEs; other countries have similar administrative divisions; and many clinical trials are multinational, particularly industry-led trials. There are many other blockers to using HSD for delivery-through-data of clinical trials. [8, 44] Trial sponsors may persist with tradition until all of these blockers have been addressed. Therefore, work is required rapidly to resolve each of these issues to make progress in streamlining the delivery of clinical trials.

## Conclusion

There is a strong need to streamline clinical trials with better use of health systems data. However, there can be a penalty to be paid for any clinical trial that needs to use a two-stage aggregate data meta-analysis approach, which cannot easily be offset without increasing sample size as a minimum. Further work is required to enable one-stage analysis.

## Acknowledgements

The salary of SBL was supported by UKRI (UK Research and Innovation; MRC (Medical Research Council) [grant number MC_UU_00004/08]. The salaries of IRW and DF were supported by UKRI [grant number MC_UU_00004/09]. The salaries of SF, SBL, MLM, MRS were supported by HDRUK: HDRUK2023.0025. MRS is employed by NHS England and contributed to this paper as part of the Data for Research and Development. This research was Powered by NHS Data.

We thank Andy Payne and Will Browne for critical comments during the drafting process.

We thank the investigators of, and all participants in, the STAMPEDE trial, German Breast Cancer study and each of the trials in the TB-IPD dataset for making data available for this project

This work uses data obtained through the WHO TB treatment individual patient data platform (TB-IPD), a collaborative initiative led by the World Health Organization (WHO), Global TB Programme and University College London (UCL).

## Contributions

Using CREDIT taxonomy

**Table:** 

**Area**	**Description**	**SMF**	**SBL**	**DF**	**IRW**	**JFT**	**CD**	**AWF**	**PQ**	**MLM**	**MRS**
**Conceptualisation**	Ideas; formulation or evolution of overarching research goals and aims.	X	X	X	X	X		X		X	X
**Data Curation**	Management activities to annotate (produce metadata), scrub data and maintain research data (including software code, where it is necessary for interpreting the data itself) for initial use and later reuse.	X		X	X						X
**Formal Analysis**	Application of statistical, mathematical, computational, or other formal techniques to analyse or synthesise study data.	X		X	X						
**Funding Acquisition**	Acquisition of the financial support for the project leading to this publication.									X	X
**Investigation**	Conducting a research and investigation process, specifically performing the experiments, or data/evidence collection.	X	X	X	X					X	X
**Methodology**	Development or design of methodology; creation of models.	X	X	X	X					X	X
**Project Administration**	Management and coordination responsibility for the research activity planning and execution.	X	X	X	X					X	X
**Resources**	Provision of study materials, reagents, materials, patients, laboratory samples, animals, instrumentation, computing resources, or other analysis tools.										
**Software**	Programming, software development; designing computer programs; implementation of the computer code and supporting algorithms; testing of existing code components.	X		X	X						X
**Supervision**	Oversight and leadership responsibility for the research activity planning and execution, including mentorship external to the core team.		X							X	X
**Validation**	Verification, whether as a part of the activity or separate, of the overall replication/reproducibility of results/experiments and other research outputs.			X	X						
**Visualisation**	Preparation, creation and/or presentation of the published work, specifically visualisation/data presentation.	X	X	X	X					X	X
**Writing – Original Draft Preparation**	Creation and/or presentation of the published work, specifically writing the initial draft (including substantive translation).	X	X	X	X					X	X
**Writing – Review & Editing**	Preparation, creation and/or presentation of the published work by those from the original research group, specifically critical review, commentary or revision – including pre- or post-publication stages.	X	X	X	X	X	X	X	X	X	X

## Data Availability

The German breast cancer data are freely available from StataCorp [21] at https://www.stata-press.com/data/r16/brcancer.dta. TB-IPD is available from the Data Access Committee of TB-IPD: https://www.ucl.ac.uk/population-health-sciences/global-health/research/research-projects/tb-ipd-platform. The STAMPEDE trial was sponsored by UCL and coordinated by the MRC Clinical Trials Unit at UCL, full details are available at the trial website: www.stampedetrial.org. [23] STAMPEDE data can be requested from https://www.mrcctu.ucl.ac.uk/our-research/other-research-policy/data-sharing/.

## Abbreviations

HIV:	Human Immunodeficiency Virus
HSD:	Health Systems Dataset
IPD:	Individual Participant Data
NSAID:	Non-Steroidal Anti-Inflammatory Drug
ONS:	Office for National Statistics
PSA:	Prostate-Specific Antigen
SDE:	Secure Data Environments
SE:	Standard Error
TB:	Tuberculosis
Var:	Variance
WHO:	World Health Organization
